# Estimated Impacts of Alcohol Control Policies on NCD Premature Deaths in Thailand

**DOI:** 10.3390/ijerph19159623

**Published:** 2022-08-04

**Authors:** Surasak Chaiyasong, Jie Gao, Kanitta Bundhamcharoen

**Affiliations:** 1 Alcohol and Health Promotion Policy Research Unit and Social Pharmacy Research Unit, Faculty of Pharmacy, Mahasarakham University, Mahasarakham 44150, Thailand; 2 International Health Policy Program, Ministry of Public Health, Nonthaburi 11000, Thailand

**Keywords:** alcohol, premature death, non-communicable disease, global target, Thailand

## Abstract

**Background:** This study aimed to assess the impacts of achieving a 10% alcohol reduction target and different alcohol policy interventions on NCD premature deaths during 2010–2025 in Thailand. **Methods:** The researchers estimated the impacts on three main NCDs: cancers, cardiovascular diseases, and diabetes. These represent two ideal scenarios, which are the target reduction and five intervention scenarios. These intervention scenarios comprise taxation with 50% price increases, a total ban on advertisements, availability restriction by shortening sales times, early psychological intervention, and combined interventions. Consumption data and mortality trends were obtained from available national data. Relative risks and intervention effects were derived from the literature. **Results:** Achieving a 10% reduction target would lead to 3903–7997 avoidable NCD deaths. Taxation was the most effective intervention, with the highest number of avoidable NCD deaths, followed by early psychological intervention, availability restriction, and an advertisement ban. A combination of these four interventions would reduce 13,286 NCD deaths among men and 4994 NCD deaths among women, accounting for 46.8% of the NCD mortality target. **Conclusion:** This study suggests using Thailand as an example for low- and middle-income countries to enhance implementation and enforcement of the recommended effective alcohol policies for achieving the global targets.

## 1. Introduction

Non-communicable diseases (NCDs) cause more than 40 million deaths worldwide every year and account for 71% of all global deaths [1]. Cancers, cardiovascular diseases, chronic respiratory diseases, and diabetes are the four main NCDs mostly accounted for in the total NCD mortality figures. In 2011, the UN General Assembly adopted a declaration that committed member states prevent and control NCDs, and countries agreed to adopting nine global targets, including an overarching target of reducing premature mortality from the four main NCDs by 25%, relative to their 2010 level, by 2025 [2]. In addition, the reduction in NCD premature mortality is included in the Sustainable Development Goals (SDGs), as the SDG target 3.4 is to reduce NCD premature mortality by a third by 2030, relative to the 2015 level, and to promote mental health and well-being [3]. 

A study estimated the contribution of achieving six risk-factor targets toward meeting the global NCD mortality target, indicating that if the risk-factor targets are achieved, the probability of dying from the four main NCDs between the ages of 30 years and 70 years will decrease by 22% among men and by 19% among women between 2010 and 2025, compared with a decrease of 11% among men and 10% among women under the so-called business-as-usual trends (i.e., projections based on current trends, with no additional action). Most of the benefits of achieving the risk factor targets will be in low-income and middle-income countries (LMICs) [4]. Furthermore, the effective pathways to achieving the SDG target have been proposed [5].

Alcohol consumption is a causal factor for more than 200 diseases and related injuries, attributing to 5.3% of premature deaths and 5.1% of the global burden of diseases and injuries [6]. The global target of alcohol consumption is set at a 10% per capita reduction in consumption by 2025 relative to its 2010 level. Therefore, the World Health Organization (WHO) has recommended a set of best-buy and cost-effective policy interventions, particularly taxation and pricing policies, marketing control, regulations on availability of alcohol, drunk-driving countermeasures, and early psychosocial interventions [7,8,9,10]. 

Thailand is a country with a low drinking prevalence of 28.4% among all Thai adults aged 15 years and older [11], with a per capita alcohol consumption of 8.3 liters of pure alcohol [6]. Over a decade, Thailand has implemented various policies and interventions according to the recommendations of the Global Strategy to reduce the harmful use of alcohol and the Global Action Plan for the prevention and control of NCDs. In 2010, Thailand launched a national alcohol policy strategy and action plan for alcohol prevention and control during the period 2011–2020. However, the enforcement of all alcohol control regulations and measures in Thailand has relied on three main legislations with administration by different ministries: Alcoholic Beverage Control Act of 2008 administered by the Ministry of Public Health, the Excise Act of 2018 (previously the Liquor Act of 1950) by the Ministry of Finance, and the Road Traffic Act of 1979 by the Ministry of Interior [12]. 

### Summary of Regulations of the Five Recommended Alcohol Policies in Thailand

In Thailand, all alcoholic beverages are taxed under a combination of specific and ad valorem taxation methods. A licensing system is established for the prevention and control of production, distribution, and sales of alcoholic beverages. Selling and drinking alcohol are restricted in certain places, such as schools, dormitories, gas stations, and public parks. The availability of alcohol is also regulated, including time of sales, day of sales, minimum legal purchase age (20 years), minimum legal drinking age (18 years), selling alcohol to intoxicated persons, and selling methods and conditions. The advertisement of alcohol is partially banned where alcohol product advertisement is banned, but corporate advertisements can be broadcasted from 10 pm to 5 am. In addition, advertising content, medium, and time are also regulated. Any price promotions are prohibited. For drunk-driving countermeasures, the blood alcohol concentration limit is at 0.5 mg%, while random breath testing and sobriety checkpoints are implemented. Screening, early interventions, and treatment of alcohol-use disorders are provided in some hospitals and healthcare units [12]. Although Thailand has many alcohol policies and regulations, alcohol consumption has slightly increased, particularly in younger and female populations. 

As alcohol consumption and its related NCD mortality rate have been targeted for a 10% reduction by 2025 [3], Thailand definitely needs more action by strengthening current alcohol control policies and regulations and developing other non-existing ones. From the researchers’ experience, there is no study in Thailand that estimates whether or not the target of a 10% reduction in alcohol consumption and corresponding avoidable causes of death is achievable by 2025. Therefore, this study has aimed to project alcohol consumption and avoidable NCD premature deaths in 2025 under various scenarios, together with assessing the effects of different alcohol policy interventions on preventing NCD premature deaths. This study might shed light on alcohol policy development in Thailand and be an example for other LMICs. To achieve the study’s objective, three specific research questions were focused upon: (1) If alcohol use was reduced by 10%, how many NCD deaths could be prevented? (2) Could NCD deaths be better prevented if the proportion of the reduction of the heavier drinking group is greater than in other groups? (3) Which alcohol policy interventions could reduce most alcohol consumption and related NCD mortalities in Thailand?

## 2. Materials and Methods

### 2.1. Study Design

The researchers estimated and modeled the impacts of reducing alcohol consumption against the global target on NCD mortality in Thailand between 2010 and 2025. It is noticed that alcohol use is a risk factor for more than 200 diseases and injuries [6]. However, this study emphasized only three NCDs as the main alcohol-attributable causes of premature deaths: cancers, cardiovascular diseases (CVDs), and diabetes. Scenarios of alcohol policy interventions were selected based upon suggestions from alcohol-policy research experts. To be consistent with the global target, this study has defined premature mortality as the probability of dying from the main NCDs between 30 and 70 years of age, in the absence of competing causes [4].

### 2.2. Estimation of Baseline Alcohol Consumption

A baseline for alcohol consumption was estimated by using individual data disaggregated by age, sex, consumption level, and beverage-specific consumption from the Tobacco and Alcohol Consumption Behavior Survey (TAC) 2011. TAC’s methodology has been described elsewhere [11]. In brief, the TAC is a nationally representative survey of the Thai population, periodically conducted by the National Statistical Office once every three years, using a two-stage stratified sampling method to collect data in all 77 provinces throughout the nation. This survey can provide estimates of drinking prevalence from national to provincial levels. It is the biggest national alcohol survey in Thailand, with a total sample size of 177,350 respondents aged 11 years old or above. However, this study used data from a total of 128,847 respondents between 15 and 70 years of age. Within the TAC2011, a beverage-specific and quantity-frequency approaches were employed to collect previous-month alcohol consumption of seven types of alcoholic beverages, being beer, white spirits (a local, colorless, and cheap spirit), whisky, wine, wine cooler/ready-to-drink, traditional fermented alcoholic beverage, and other alcoholic beverages such as medicinal spirits or Chinese alcohol.

The researchers computed both total and average alcohol consumption expressed as a gram of ethanol intake per day (g/day). As different consumption levels have different risks of diseases and mortality, drinkers were classified into three groups according to the WHO risk drinking level definition, including high/heavy (>60 g/day among men or >40 g/day among women), moderate (>40 to 60 g/day among men or >20 to 40 g/day among women), and low/light (1 to 40 g/day among men or 1 to 20 g/day among women) [13]. (see percentages of Thai drinkers in these 3 risk-drinking categories by age and sex in Supplementary Figures S1 and S2).

During the modeling period, the researchers assumed that the alcohol use remained stable at the baseline level. The decrease in alcohol use would be hypothetically recognized as the estimated benefit of alcohol policy interventions toward the alcohol reduction target [4].

### 2.3. Scenarios

#### 2.3.1. Scenarios for Alcohol Consumption Reduction Target

To answer the first two research questions, two ideal scenarios with different alcohol reductions between three drinking groups were conducted. Ideal Scenario One is a proportional 10% reduction in alcohol consumption in each of the three groups, which are high-risk, moderate-risk, and low-risk drinkers. Ideal Scenario Two represents disproportional reductions of 20%, 10%, and 5% among the drinking groups of high-risk, moderate-risk, and low-risk drinkers, respectively.

#### 2.3.2. Effects of Policy Interventions

The third research question aimed to compare the impacts of multiple alcohol policy interventions. Out of the best buys and other cost-effective policy interventions recommended by the WHO [10,14], this study emphasized taxation, regulating the availability of alcohol, advertising bans, and brief interventions. Drinking-and-driving countermeasures were excluded because they were highly associated with traffic accidents and injuries rather than the NCDs. The effects of these alcohol policy interventions were derived from international studies unless Thai studies were available. Given these different interventions, five intervention scenarios were analyzed in this study as follows. 

##### Taxation

The price of alcohol is inversely associated with both consumption levels and the prevalence of alcohol-related harmful effects [15,16]. Moreover, it is positively associated with increasing excise tax rates, resulting in, for example, higher retail prices. The theory of consumer demand explains that increasing alcohol prices reduces alcohol consumption. The response to price changes resulting from a 1-percent change in price is called the price elasticity of demand for alcohol. This research obtained price elasticity data from a study using data from the International Alcohol Control Policy (IAC) Study in Thailand, with −0.22 (95% CI: −0.38, −0.06) for beer, −0.71 (−1.33, −0.71) for white spirits, −0.20 (−0.36, −0.04) for whisky, and −0.49 (−0.60, −0.38) for all types of alcoholic beverages [17]. Thus, when the price of alcoholic beverage types individually increases by 10%, the consumption of beer, white spirits, whisky, and all types of alcohol then decreases by 2.2%, 7.1%, 2.0%, and 4.9%, respectively.

In Thailand, rates of alcohol taxation have been revised many times over the years, resulting in the raising of alcoholic-beverage prices between 5% and 25%. As an ambitious but feasible strategy, the researchers adopted a scenario of a 50% price increase, as proposed for all participating countries [8], to simulate individuals’ consumption changes in all alcoholic-beverage types. 

##### Advertising Ban

Advertisement is the most used marketing activity to create an alcohol-drinking atmosphere and encourage targeted groups to use alcohol. Since 2008, Thailand has conducted partial statutory restrictions on alcohol advertisement [18] that require advertisements of alcoholic beverages to avoid inducing people to consume and has enacted restrictions on the display of the product or packaging. A scenario of a total ban on all types of alcohol-related advertisement was set up to comprehensively ban alcohol advertising.

An effect of the proposed total ban of alcohol advertisement in Thailand was expected to reduce the consumption level by 3% (95%CI: 1%, 6%) in accordance with the previous studies reporting that increasing alcohol marketing control reduced drinking volume per capita [8,18].

##### Restricting Physical Availability of Alcohol

A restriction on the physical availability of alcohol increases the economic and opportunity costs of obtaining alcohol; alcoholic beverages can be sold every day from 10 a.m. to 2 p.m., and 5 p.m. to midnight, thus preventing vulnerable and high-risk groups from easily accessing alcohol [19]. Common options of availability restriction are regulating the licensing systems of outlets, controlling outlet density, and regulating the days and hours of retail sales. 

Compared to other approaches, restricting business hours and licensing outlets is more effective [8,20]. Considering that Thailand has already applied the licensing system, the researchers tested the effects of shortening Thai business hours. Due to the lack of works in the literature in low- and middle-income countries, this study used figures from a study based on high-income countries [21], entailing that reducing one day of sales decreases consumption per capita by 3.4% (95%CI: 2.7, 4.1). The reduction in business hours used in this study related to the decrease in either times or days of sale for one day a week. 

##### Early Psychological Intervention

Early psychological intervention has been proven to be effective in terms of alleviating heavy drinking behaviors [22,23]. It is composed of several steps, such as screening people involved with hazardous/harmful use of alcohol (moderate/heavy drinking levels), providing consulting and medical interventions, and evaluating outcomes at follow-up. Previous studies reported that the early psychological intervention reduced 3.6 drinks per week among heavy drinkers or roughly equivalent to 13.7 g per week [18,23]. This study assumed that 100% of moderate and heavy drinkers were given the national screening and early intervention.

##### Combined Interventions

This scenario was a combination of all four proposed interventions together, including taxation resulting in 50% price increases, a total ban of alcohol advertisements, shortening sales times one day per week, and combining these with early psychological intervention.

### 2.4. Modeling Alcohol Consumption Reduction and NCD Mortality

The modeling used in this study was composed of two segments: policy-to-consumption and consumption-to-harm. These used were to estimate changes of alcohol consumption from a baseline and project premature mortality out of targeted NCDs until 2025 as impacts of the five proposed alcohol policy interventions.

#### 2.4.1. Policy-to-Consumption Part

This study employed individual-level data of TAC2011 to estimate alcohol consumption of the Thai population in various scenarios of alcohol policy interventions. Changes in alcohol consumption were the key outputs derived in this part by comparing estimated alcohol consumption after alcohol policy interventions with the baseline alcohol consumption. In doing so, intervention effects were computed for individuals by multiplying their consumption values with intervention-effect values. For the taxation scenario, the individual consumption of specific alcoholic beverage types was computed firstly, and then the total consumption was calculated. Modeling estimates of consumption changes due to alcohol policy interventions were conducted by using Stata, version 15. Changes in the prevalence of at-risk drinking levels were used to examine the impacts on mortality in a consumption-to-harm part.

#### 2.4.2. Consumption-to-Harm Part

The impacts of alcohol consumption changes were estimated as premature deaths based on the model of burden of disease in Thailand. Three major NCDs included in this study were identified based on the ICD codes (see Supplementary Table S1). The 2001–2016 Vital Registration datasets were employed to identify disease outcomes and project mortality rate. Alcohol attributable fractions (AAFs) were applied to estimate premature deaths due to the three main NCDs. In this section of the study, all statistical analyses were performed by using Stata v.15, and spreadsheets were used for calculation.

#### 2.4.3. Alcohol-Attributable Fractions

The researchers used AAFs to estimate the proportion of the health burden that could be eliminated by stopping or reducing alcohol use so that one could link alcohol consumption data with death information. The calculation of AAFs adapted based on the CRA methodology, a proportions-shift method [24,25], is as follows:

(1) $$AAFs=\frac{\sum_{i}^{n}P_{i}RR_{i}-\sum_{i}^{n}P_{i}^{2}RR_{i}}{\sum_{i=1}^{n}P_{i}RR_{i}}$$

where *P_i_* represents the proportion of population at exposure level *i*, $P_{i}^{2}$ represents the proportion of population at exposure level *i* after the interventions of the study scenarios, and *RR_i_* represents the relative risks of alcohol use at exposure level *i*. 

For the calculation of combined AAFs for a number of interventions, the study used the following formula [26]: AAF_combined_ = 1 − (1 − AAF_policy1_) × (1 − AAF_policy2_) × (1 − AAF_policy3_) × (1 − AAF_policy4_). 

By multiplying AAFs with premature mortality rates (mortality rates of people aged between 30 years and 70 years) associated with certain diseases, this research calculated the avoidable premature mortalities caused by alcohol use control.

#### 2.4.4. Relative Risks for Targeted Diseases

This study used the relative risks (RRs) of three targeted NCDs, namely cancers, CVDs, and diabetes, but not chronic respiratory diseases, because there is no direct association with alcohol use [27]. All the relative risks of each disease were extracted from previous epidemiological studies with pooled meta-analyses on cancers [28,29], CVDs [28,30,31,32], and diabetes [33,34] (see more details on the RRs and ICD-Codes in Supplementary Table S1). 

Although some studies reported protective effects of moderating drinking toward preventing CVDs and diabetes [35,36], a recent study stated that the existing evidence fails to approve a safe level of alcohol drinking without any negative impacts [37]. Considering that this study only focuses on the detrimental effects of alcohol use, all the positive effects (RR < 1) from the literature reviews were replaced with no effects (RR = 1).

#### 2.4.5. Premature Mortality

This study has adopted the probability of dying between the age of 30 and 70 years to represent premature mortality [4]. This indicator creates comparable results by eliminating the influence of other competing causes on mortality, such as communicable disease and suicide.

Vital registration data between 2001 and 2016 were first divided by five-year age intervals and sex. Due to the volatility of data over time, an exponential smoother was applied at first for the sake of adding more weight to more recent data. Given the nature of count data, the researchers conducted Poisson regression for the extrapolations. After that, life table methods [38] were used to generate the probability of the affected population dying at ages from 30 to 70 years. At the end, the study applied the data to an estimated population from 2010 to 2025 to estimate the absolute numbers of avoidable deaths. The population information was extracted from a report by the Thai Ministry of Public Health [39].

#### 2.4.6. Lag and Latency Time

Alcohol-related NCDs are partly caused by the accumulated effects of alcohol use. Thereby, the adverse effects of drinking may be delayed or last for a period for some diseases [40]. For CVDs and diabetes, the changes in incidences appear immediately after reducing alcohol consumption, while for cancers, the lag time between changes in alcohol consumption and the changes in harm effects was set as one year [40,41]. With a latency period of 15 years, all changes in harmful effects, either increasing or declining during the study period, were estimated with a linear function.

### 2.5. Analysis

All statistical analyses were conducted by using Stata v.15. Sampling weights were applied for the analysis of TAC2011 to take into account surveyed complex designs and provide nationally representative estimates. Spreadsheets were used to project alcohol consumption and mortality rates over the study period and to calculate avoidable deaths of the targeted NCDs due to various alcohol policy interventions. This study estimated the potential total number of NCD deaths in achieving the global target of 25% reduction (the 25 × 25 target) among men and women. There are 95% confident intervals (95% CIs) of the estimated numbers of NCD deaths that were reported for all scenarios.

## 3. Results

**Figure 1:**
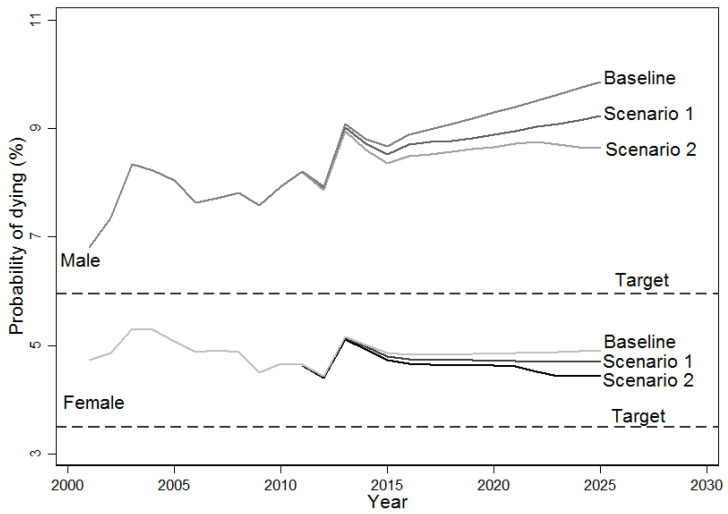
Probability of dying out of targeted NCDs among Thais aged between 30 and 70 by sex for baseline (no intervention) and ideal scenarios (10% alcohol use reduction).

At the baseline of this modeling, among Thais aged between 15 and 70 years, more men consumed alcohol than women. In general, light drinkers, moderate drinkers, and heavy drinkers accounted for 37.9%, 2.9%, and 5.0% among men and 5.5%, 0.5%, and 0.6% among women, respectively (see more detail in Supplementary Figure S1). When modeling premature deaths from the three NCDs attributable to alcohol over the study period was undertaken, it was found that the ideal scenarios of 10% reduction in alcohol consumption would lower the probability of dying out of targeted NCDs compared with the baseline (no intervention) scenario. Scenario Two (with expected high reduction in heavy drinkers) hypothetically performed better than Scenario One in terms of reducing the probability of dying out of the targeted NCDs for both sexes, although neither scenario could attain the global target of 25% reduction in avoidable premature probability of dying (Figure 1). For men, the probability of dying increased from 7.9% in 2010 to 9.9% in 2025 in the baseline scenario, and there was a 11.1% and 21.6% increase of the global target in Scenarios One and Two, respectively. Compared to men, women had a small increase in the expected probability of dying, from 4.7% in 2010 to 4.9% in 2025 in the baseline scenario, with 7.9% and 18.2% achieving the global target ideal in Scenarios One and Two, respectively.

To achieve the 25% NCD death reduction (the 25 × 25 target), the total numbers of avoidable NCD deaths related to alcohol would be 39,093 deaths. Of these, 26,150 deaths were men, and 12,943 deaths were women. The alcohol target of 10% reduction in alcohol use would reduce premature NCD deaths, in total, between 3930 and 7997 deaths, which account for 2.5−5.1% of total NCD deaths (or 10.1−20.5% of the 25 × 25 target). This study revealed that more alcohol use reduction in heavy drinker groups would result in more avoidable NCD deaths. For scenarios of alcohol policy interventions, all four single interventions could reduce the numbers of premature deaths where taxation was most effective. Taxation with a 50% price increase would reduce 10,906 deaths among men and 3423 deaths among women, accounting for 41.7% of an expected total number of avoidable NCD deaths among men and 26.5% of that among women, respectively. Furthermore, combined interventions of these four strategies would lead to the most avoidable NCD deaths, with 13,286 deaths (50.8%) among men and 4994 deaths (38.6%) among women (Table 1). 

## 4. Discussion

This is the first Thai study on the impacts of alcohol policy interventions in response to achieving the NCD mortality target by investigating the impacts of reducing alcohol use on avoiding premature deaths, i.e., cancers, cardiovascular diseases, and diabetes, from 2010 to 2025 and examined contributions to the target achievement of various alcohol policy interventions in Thailand.

Note: Baselines represent the trends of probability of dying if alcohol consumption levels stay at the 2010 level. Scenario One assumes a 10% relative reduction of alcohol consumption amount among all three consumption levels. Scenario Two assumes a 20% reduction of drinking amounts among heavy drinkers, 10% with moderate drinkers, and 5% with light drinkers. Targets represent the global target for reducing premature deaths; that is, there is a 10% relative reduction in the probability of dying from related NCDs compared with the 2010 level. 

Achieving a 10% reduction in alcohol consumption would lead to 3903–7997 avoidable NCD deaths, depending on how much reduction takes place between drinker groups. This would contribute to reducing the targeted NCD mortality by 2025 between 2.5 and 5.1% in Thailand. A previous study projected that achieving all six risk factor targets, including the alcohol target, will lead to a total reduction of NCD mortality by 22% among men and 19% among women; however, it is not sufficient to meet the 25 × 25 target [4]. Thus, the study suggests that not only a more ambitious target should be considered, but the implementation and enforcement of effective and cost-effective strategies should also be determined to achieve the targets [7,8,10]. In comparing the projected probability of dying under two ideal scenarios of 10% alcohol use reduction in the present study, more reduced alcohol consumption among heavy drinkers is found to bring more benefits in avoiding NCD deaths, as a high proportion of alcohol is consumed in harmful drinking [42]. This can be explained by the fact that that heavier drinking is more likely to bring disease and death more than light-to-moderate drinking [43,44]. This finding indicates that, to reduce more alcohol consumption and NCD mortality, strategies to prevent and control heavy drinking are critical.

There are several studies modeling impacts of alcohol policy interventions in terms of reduced alcohol consumption, diseases and probability of dying, avoidable premature death, and the burden of diseases as affecting disability-adjusted life years [8,45,46,47]. This study models the impacts of various alcohol policy interventions based on available Thai data and the international literature. However, research on best-buy alcohol interventions in low- and middle-income countries is limited [48]. Thus, this study retrieved most of the intervention effects from the international literature originating in high-income countries. 

Based on the best available country-specific data, taxation is the most effective intervention with the highest number of avoidable NCD premature deaths, followed by early psychological interventions, availability control, and advertisement bans. The order of effective interventions in this study is similar to the order of interventions reported by Chisholm et al., which modeled the effects, costs, and cost-effectiveness of five alcohol control strategies for upper-middle- and high-income countries, including Thailand [8]. 

In this study, levying taxes on alcoholic beverages to result in a 50% relative price increase could significantly avoid more premature deaths than other single alcohol policy interventions. This finding is consistent with previous studies which indicate that alcohol taxation is one of the most effective policies to reduce alcohol consumption and related harmful effects [7,8,45,46]. According to the alcohol consumption profile, three types of alcoholic beverages (i.e., beer, white spirits, and whiskey) account for more than 95% of alcohol consumption in the Thai population. This suggests that the three beverage types are targets for taxing alcohol-related NCD mortality. Although drinkers may switch to other types of alcoholic beverages, they preferred it if prices of their current consumed beverages increased, and this substitution effect may be very low when prices of all alcoholic beverage types increase similarly. 

Despite taxation policies, early psychological interventions are regarded as the most effective policy on avoiding premature deaths compared with advertisement bans and availability restrictions. Early psychological interventions are mainly targeted at drinkers with dependence and thus could effectively mitigate the probability of developing chronic diseases and dying prematurely among those who are already at risk. To effectively implement this policy option, a number of healthcare providers with knowledge and skills related to these psychological interventions are needed, especially at primary care facilities. For example, a screening and brief intervention program could decrease alcohol and substance misuse while patients are in primary care [49]. As this scenario assumed a 100% coverage of the targeted population, a system for the management of alcohol-use disorders should be organized to be more accessible to the public.

The two recommended best-buy interventions, controlling physical availability and the marketing of alcohol, could reduce a number of NCD premature deaths, but not as many as potentially affected by taxation and psychological interventions. These two interventions are a population-wide intervention similar to taxation, but their effectiveness on reducing alcohol consumption and related harm is not much reported in low- and middle-income countries [48]. They also have effects on reduced initiation rates of alcohol use [50] which may reduce the propensity of drinking rather than the intensity of drinking. Hence, if the effect on drinking initiation would be included, impacts on premature death would be increased. Regarding current regulations on the availability of alcohol in Thailand, alcoholic beverages can be sold every day from 10 a.m. to 2 p.m., and 5 p.m. to midnight, while and their related advertisements are partially banned. The scenarios on shortening the hours or days of sales and a total ban of alcohol advertisement have been proposed to prevent and reduce alcohol consumption among young people. 

As to a combination of multiple interventions, little is known about its effect. This study used a multiplicative approach to combining intervention effects with the assumption that each intervention always reduces the prevalence of alcohol consumption, and later the probability of dying by NCDs by the same proportion, whether it is implemented on its own or in combination with other interventions [45]. In addition, the figures are lower than the sum up of avoidable deaths out of all included interventions. Moreover, there are likely to be some overlapping effects among interventions that would reduce the overall effects of combined interventions. As a result of this approach, a combination of the four interventions would reduce a greater number of NCD premature deaths than each single intervention, with 13,286 avoidable NCD deaths among men and 4994 avoidable NCD deaths among women. This total reduction would account for 46.8% of the NCD mortality target. Therefore, this policy option should be the highest priority for implementation to reduce alcohol use and related harmful effects in terms of NCDs. This would be one of the pathways to achieve the SDG target 3.4—to reduce NCD premature mortality by a third by 2030, relative to the 2015 level, and to promote mental health and well-being. 

The results of this modeling indicate that the implementation of any recommended effective interventions during 2010–2025 would reduce alcohol consumption and NCD premature deaths. Although since 2010 Thailand has introduced several laws and regulations to prevent and control alcohol consumption and related harmful effects [12], some of the recommended effective interventions were implemented and enforced. For instance, in 2015, the government announced the prohibition of selling and drinking alcohol at governmental workplaces, public transportation stations, and on trains; announced a ban on selling alcohol on five national religious days; and established zoning areas prohibiting alcohol sales around schools. In 2017, the alcohol taxation system was restructured, and excise tax rates were slightly increased. The estimates of alcohol consumption per capita slightly increased from 6.19 L of pure alcohol (LPA) in 2010 to 7.33 LPA in 2017, and to 6.86 LPA in 2019 [12].

As the Thai National Alcohol Policy Strategy reached its termination in 2020, the Joint Assessment Team was commissioned to evaluate the implementation of the strategy. With regard to recommendations from the Assessment Team, there are measures that Thailand has implemented and will further increase its intensity, such as participation of local communities and strict enforcement of relevant legal measures. Example measures would be the prohibition of alcohol sales to individuals under 20 years old, a reduction of the number and density of alcohol outlets, a ban on all forms of alcohol advertisement (particularly via digital media), and the prioritization of participation of local communities in promoting legal compliance and creating models for festivals that are alcohol-free [51]. 

This study has several limitations. Firstly, the baseline data of alcohol consumption of the Thai population were derived from the Tobacco and Alcohol Consumption Behavior Survey of 2011 only, due to no data being available for 2010. In regard to the survey data, alcohol consumption is underreported compared with sales data [52]. Secondly, this study included only the three main NCDs, resulting in some alcohol-related diseases and injuries to fall outside of the parameters of the study. Thus, recommended effective interventions such as drunk-driving countermeasures, which had strong effects on road traffic accidents and mortality, were not included, as well. Thirdly, due to the poor quality of the cause-of-death statistics in Thailand [53], the researchers’ estimates may not reflect the real number of targeted NCD deaths. This study, however, has focused upon, and predicted premature deaths in terms of changes from the baseline. This reflects the critical role of the quality of mortality data, both in terms of completeness of reporting and the quality of cause-of-death statistics. Fourthly, research on the effectiveness of alcohol policy interventions in low- and middle-income countries is lacking, especially in the best-buy interventions [48]. Therefore, available evidence from high-income countries was applied, although there might be differences of alcohol environments and cultures between these groups of countries. Lastly, this study modeled the impacts of alcohol policy interventions based on the best available data, especially country-specific data, and several assumptions. Hence, the findings of this study should be used with awareness of its limitations.

Although this research uses Thai data, its findings have international implications for LMICs which have consumption and mortality profiles similar to Thailand’s. Future research should add more diseases and conditions related to alcohol in its modeling. Costs and cost-effectiveness analyses of alcohol policy interventions should also be studied.

## 5. Conclusions

Achieving a 10% reduction target would lead to a number of NCD deaths being avoided, as a reduction of heavier drinkers would also reduce NCD mortality rates. Taxation is the most effective intervention, followed by early psychological intervention, availability control, and advertisement bans. If all alcohol policy interventions were implemented together, premature deaths from the three main NCDs—cancers, cardiovascular diseases, and diabetes—would be reduced to half of the 25 × 25 NCD global target. This study suggests Thailand as an example for low- and middle-income countries to use to enhance the implementation and enforcement of the recommended effective alcohol policies.

## Figures and Tables

**Table 1:** Avoidable NCD deaths and percent in achieving the global target in 2025 of the study scenarios.

Scenarios	Avoidable NCD Deaths			
	Male		Female	
	No. (95%CI ^a^)	% ^b^	No. (95%CI ^a^)	% ^b^
**Total number of NCD deaths in achieving the global target (25% reduction)**	**26,150**	**100%**	**12,943**	**100%**
**Ideal scenarios of 10% alcohol consumption reduction**				
Ideal Scenario 1 ^c^	2907	11.1%	1023	7.9%
Ideal Scenario 2 ^d^	5639	21.6%	2358	18.2%
**Intervention scenarios**				
Taxation (50% price increase)	10,906 (10,567–11,126)	41.7%	3423 (3176–3654)	26.5%
Advertisement ban	776 (664–1149)	3.0%	303 (118–489)	2.0%
Restricting availabilities	904 (2178–2408)	3.5%	1387 (1194–1584)	2.3%
Psychological interventions	2290 (601–952)	8.8%	258 (117–406)	10.7%
Combined interventions ^e^	13,286 (12,664–13,884)	50.8%	4994 (4270–5670)	38.6%

^a^ This 95% CI is the 95% confidence interval, which is extracted from simulations of alcohol consumption levels and then translated into avoidable deaths. ^b^ Global target represents a 25% relative reduction of premature mortalities from targeted alcohol-related NCDs to their 2010 levels by 2025. ^c^ Ideal Scenario One represents a 10% relative reduction of the consumption amount among heavy drinkers, moderate drinkers, and light drinkers, respectively. ^d^ Ideal Scenario Two represents a 20% relative reduction of the consumption amount among heavy drinkers, 10% among moderate drinkers, and 5% among light drinkers. ^e^ Combined interventions comprise a taxation with a 50% price increase, shortening sales times one day a week, a total ban of alcohol advertisements, and early psychological intervention.

## Data Availability

The Tobacco and Alcohol Consumption Behavior Survey and Vital Registration data can be retrieved from the National Statistical Office. The data used in this study are available from the authors upon reasonable request and with permission of the National Statistical Office.

## Supplementary Materials

The following supporting information can be downloaded at https://www.mdpi.com/article/10.3390/ijerph19159623/s1. Figure S1: Proportions of drinkers consuming seven types of alcoholic beverages by sex, estimated from the Tobacco and Alcohol Consumption Behavior Survey 2011. Figure S2: Percentages of drinking categories in Thai population by sex and age groups, estimated from the Tobacco and Alcohol Drinking Behavior survey 2011. Table S1: Categories of non-communication diseases (NCDs), ICD codes, relative risks (RRs) by NCDs, and groups of drinkers.
