# Supplementary material for: Estimated Impacts of Alcohol Control Policies on NCD Premature Deaths in Thailand
Source: Int J Environ Res Public Health. 2022 Aug 4;19(15):9623. doi: 10.3390/ijerph19159623 (PMC9368053; doi:10.3390/ijerph19159623)
Supplement: Supplementary file 1 [file ijerph-19-09623-s001.zip › ijerph-1811776-supplementary.pdf]

Supplementary Materials

Figure S1. Proportions of drinkers consuming 7 types of alcoholic beverages by sex, estimated from the Tobacco and Alcohol Consumption Behavior Survey 2011

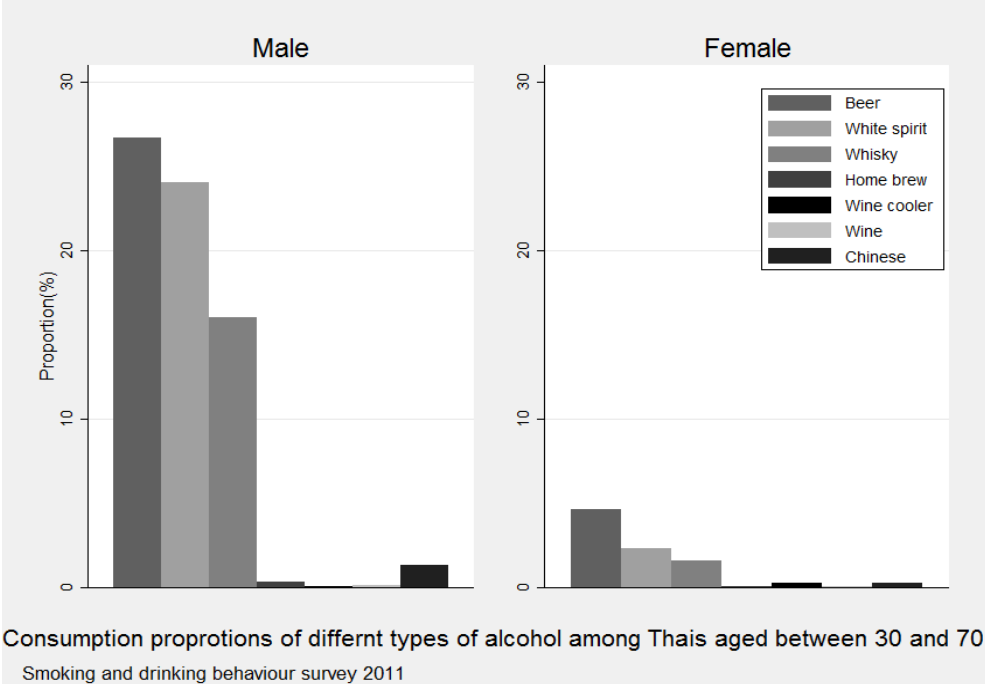

Note: The proportions are weighted estimates based on a total sample of 128,847 TAC respondents.

Figure S2. Percentages of drinking categories in Thai population by sex and age groups, estimated from the Tobacco and Alcohol Drinking Behavior survey 2011

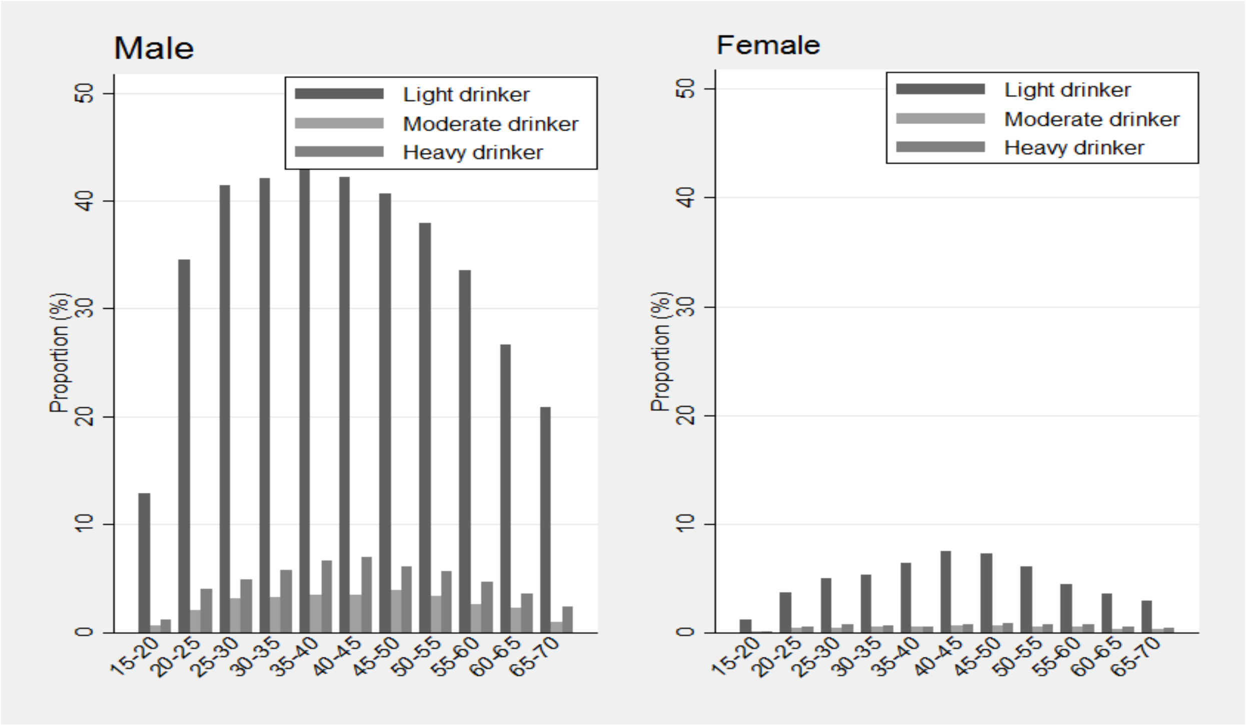

Note: Definition of risk drinking level (WHO, 2000): high/heavy is >60 g/day in men or >40 g/day in women, moderate is >40 to 60 g/day in men or >20 to 40 g/day in women, and low/light is 1 to 40 g/day in men or 1 to 20 g/day in women. The proportions are weighted estimates based on a total sample of 128,847 TAC respondents.

Table S1. Categories of non-communication diseases (NCDs), ICD codes, Relative Risks (RR) by NCDs and groups of drinkers.

NCDs categories	Diseases (ICD code)	Non-drinker	RRs by Drinker group			Sources
			High	Moderate	Low-	
Cancers	Mouth oropharynx (C0-C10)	1	5.13	1.83	1.13	(Bagnardi *et al.*, 2015)
	Oesophageal (C15)	1	3.59	1.93	1.39	(Corrao *et al.*, 2004)
	Colon and rectum (C18-C21)	1	1.44	1.17	1.00	(Bagnardi *et al.*, 2015)
	Liver (C22)	1	2.07	1.08	1.00	(Bagnardi *et al.*, 2015)
	Pancreas (C25)	1	1.19	1.03	1.00	(Bagnardi *et al.*, 2015)
	Breast (C50)	1	F=1.61	F=1.23	F=1.04	(Bagnardi *et al.*, 2015)
Cardiovascular diseases	Ischaemic heart disease (I20-I25)	1	M=1.04; F=1.23	M=1.04; F=1.41	M=1.04; F=1.03	(Roerecke & Rehm, 2014)*
	Ischaemic stroke (I63-I67)	1	1.04	1.04	1.04	(Patra *et al.*, 2010)*
	Haemorrhagic stroke (I60-I62, I69)	1	M=1.11; F=1.17	M=1.11; F=1.17	M=1.11; F=1.17	(Patra *et al.*, 2010)*
	Hypertensive heart disease (I11, I13)	1	4.15	2.04	1.43	(Corrao *et al.*, 2004)
	Atrial fibrillation and flutter (I48)	1	M=1.53; F=2.18	M=1.25; F=1.17	M=1.02; F=1.00	(Samokhvalov *et al.*, 2010)
Diabetes	Diabetes (E10-E14)	1	M=1.01; F=1.02	M=1.00; F=1.00	M=1.00; F=1.00	(Baliunas *et al.*, 2009)

Note: M=male, F=female, * There are RRs for all drinkers but no stratified RRs by drinker group.

Although some studies reported protective effects of moderating drinking toward preventing cardiovascular diseases and diabetes (Di Castelnuovo *et al.*, 2006; Ronksley *et al.*, 2011), a recent study stated that the existing evidence fails to approve a safe level of alcohol drinking without any negative impacts (GBD 2016 Alcohol Collaborators, 2018). Considering that this study only focuses on the detrimental effects of alcohol use, all the positive effects (RR < 1) from the literature reviews were replaced with no effects (RR = 1).

References

Bagnardi, V.; Rota, M.; Botteri, E.; Tramacere, I.; Islami, F.; Fedirko, V.; Scotti, L.; Jenab, M.; Turati, F.; Pasquali, E.; et al. Alcohol consumption and site-specific cancer risk: A comprehensive dose-response meta-analysis. *Br. J. Cancer* **2015**, *112*, 580–593.

Corrao, G.; Bagnardi, V.; Zambon, A.; La Vecchia, C. A meta-analysis of alcohol consumption and the risk of 15 diseases. *Prev. Med.* **2004**, *38*, 613–619.

Patra, J.; Taylor, B.; Irving, H.; Roerecke, M.; Baliunas, D.; Mohapatra, S.; Rehm, J. Alcohol consumption and the risk of morbidity and mortality for different stroke types--a systematic review and meta-analysis. *BMC Public Health* **2010**, *10*, 258.

Roerecke, M.; Rehm, J. Alcohol consumption, drinking patterns, and ischemic heart disease: A narrative review of meta-analyses and a systematic review and meta-analysis of the impact of heavy drinking occasions on risk for moderate drinkers. *BMC Med* **2014**, *12*, 182.

Samokhvalov, A.V.; Irving, H.M.; Rehm, J. Alcohol consumption as a risk factor for atrial fibrillation: A systematic review and meta-analysis. *Eur. J. Cardiovasc. Prev. Rehabil.* **2010,** *17*, 706–712.

Baliunas, D.O.; Taylor, B.J.; Irving, H.; Roerecke, M.; Patra, J.; Mohapatra, S.; Rehm, J. Alcohol as a risk factor for type 2 diabetes: A systematic review and meta-analysis. *Diabetes Care* **2009**, *32*, 2123–2132.

Howard, A.A.; Arnsten, J.H.; Gourevitch, M.N. Effect of alcohol consumption on diabetes mellitus: A systematic review. A*nn. Intern. Med.* **2004**, *140*, 211–219.

Di Castelnuovo, A.; Costanzo, S.; Bagnardi, V.; Donati, M.B.; Iacoviello, L.; de Gaetano, G. Alcohol dosing and total mortality among men and women: An updated meta-analysis of 34 prospective studies. *Arch. Intern. Med.* **2006**, *166*, 2437–2445.

Ronksley, P.E.; Brien, S.E.; Turner, B.J.; Mukamal, K.J.; Ghali, W.A. Association of alcohol consumption with selected cardiovascular disease outcomes: A systematic review and meta-analysis. *BMJ* **2011**, *342*, d671.

GBD 2016 Alcohol Collaborators. Alcohol use and burden for 195 countries and territories, 1990–2016: A systematic analysis for the Global Burden of Disease Study 2016. *Lancet* **2018**, *392*, 1015–1035.
